# An Alternative Macrophage Activation Pathway Regulator, CHIT1, May Provide a Serum and Synovial Fluid Biomarker of Periprosthetic Osteolysis

**DOI:** 10.1007/s11420-017-9598-9

**Published:** 2017-12-26

**Authors:** Samir K. Trehan, Lester Zambrana, Jonathan E. Jo, Ed Purdue, Athanos Karamitros, Joseph T. Nguyen, Joseph M. Lane

**Affiliations:** 1 Department of Orthopaedic Surgery, Hospital for Special Surgery, 535 East 70th Street, New York, NY 10021 USA; 2 Osteolysis Research Laboratory, Hospital for Special Surgery, New York, NY 10021 USA; 3 Department of Orthopaedics, 251 Hellenic Air Force and Veterans Hospital, Athens, Greece; 4 Healthcare Research Institute, Hospital for Special Surgery, New York, NY 10021 USA

**Keywords:** biomarker, osteolysis, revision total hip replacement, aseptic loosening

## Abstract

**Background:**

Periprosthetic osteolysis (PPO) is a frequent indication for total hip replacement (THR) failure. Currently, PPO diagnosis occurs in advanced stages that often necessitate complex revisions due to bone loss. PPO biomarkers could facilitate earlier diagnosis. Alternative macrophage activation pathway regulators, chitotriosidase (CHIT1) and CC chemokine ligand 18 (CCL18), have increased periprosthetic expression in patients undergoing revision THR for osteolysis. We hypothesized that synovial fluid and serum levels of CHIT1 and CCL18 would be increased in patients undergoing revision THR for PPO versus controls without osteolysis.

**Methods:**

In this prospective case-control study, 60 patients undergoing revision metal-on-polyethylene THR at Hospital for Special Surgery were screened preoperatively from January 2013 to December 2014. Twenty “osteolysis” patients who underwent revision for PPO (based on imaging and operative reports) and 10 “control” patients (with stable implants) who underwent revision for recurrent dislocation or a mechanical etiology were included. Among osteolysis and control patients, 11/20 and 4/10 were male; average age was 68 and 63 years, respectively; 9/20 and 3/10 had cemented femoral components; and average implant longevity was 15 and 5 years, respectively. Preoperative serum and intraoperative synovial fluid samples were collected. CHIT1 and CCL18 were quantified via enzyme-linked immunosorbent assay. Significance was assessed via nonparametric Mann-Whiney *U* test.

**Results:**

CHIT1 was significantly increased in both synovial fluid (3727 versus 731 nanomoles [nM]) and serum (98 versus 39 nM) in the osteolysis versus control patients. CCL18 levels were also significantly increased in osteolysis versus control patients’ synovial fluid (425 versus 180 nM) but not their serum.

**Conclusions:**

In this prospective case-control study, CHIT1 was identified as a novel synovial fluid and serum biomarker of PPO. CHIT1 expression is induced during macrophage activation in response to wear debris. CHIT1 monitoring may facilitate early diagnosis of THR PPO. Furthermore, CHIT1 may represent a novel therapeutic target for PPO.

**Electronic supplementary material:**

The online version of this article (10.1007/s11420-017-9598-9) contains supplementary material, which is available to authorized users.

## Introduction

Despite advances in implant manufacturing, wear-related periprosthetic osteolysis (PPO) remains one of the most frequent indications for total hip replacement (THR) failure and revision [19]. Data from the 2005 Nationwide Inpatient Sample, a cross-sectional sample of 1000 hospitals, revealed that PPO directly accounted for 10% of revision THRs, but an additional 43% of revision THRs were being performed for mechanical reasons likely contributed to by PPO [19]. Beck et al. estimated that out of a projected 100,000 revision THRs in the USA in 2030, up to 50,000 would be for PPO [3]. The underlying clinical issue is that the diagnosis of PPO is frequently delayed due to lack of symptoms and the difficulty associated with radiographic detection. Delayed diagnosis may necessitate complex revision surgeries due to severe bone loss. Although advanced imaging techniques, such as computed tomography (CT) and magnetic resonance imaging (MRI), are more accurate at diagnosing early stage PPO, these techniques are not routinely employed in the asymptomatic postoperative THR patient due to limitations in cost, time, and radiation exposure. For these reasons, PPO biomarker research has been performed to potentially facilitate routine screening and earlier diagnosis.

The pathophysiology of PPO has been previously investigated in vitro and in vivo. One of the central cellular players in this process is the macrophage [23]. The classical macrophage activation pathway involves the release of pro-inflammatory cytokines, including tumor necrosis factor-α and interleukin (IL)-6, in response to particulate wear debris phagocytosis by macrophages [22]. The biologic response to wear debris is dependent on several factors, such as the number of particles, particle size, particle surface morphology, and rate of particle release [14]. These inflammatory cytokines result in an imbalance of osteoclast regulators (such as osteoprotegerin [OPG] and receptor activator of nuclear factor-κB ligand [RANKL]) leading to increased osteoclast activation, bone resorption, and PPO [14]. However, an alternative macrophage activation pathway has also been described, in which there is progressive upregulation of a distinct set of inflammatory regulators in response to increasing wear debris and macrophage differentiation over time [17, 21, 22]. The roles of these mediators, including chitotriosidase (CHIT1) and CC chemokine ligand 18 (CCL18), in the progression of PPO and other disorders characterized by chronic macrophage dysfunction, are the current focus of intensive research [27].

Based on this understanding of PPO pathophysiology and the clinical need for earlier diagnosis, previous studies have investigated PPO biomarkers [1, 2, 7, 10, 12, 18, 24, 28]. Previously studied serum biomarkers include RANKL, OPG, C-reactive protein, IL-1β, bone alkaline phosphatase, osteocalcin, prostaglandin E2, matrix metalloproteinase-1, transforming growth factor-β, and tartrate-resistant acid phosphatase 5b [26]. Interestingly, few studies have evaluated mediators of the alternative macrophage activation pathway as potential PPO biomarkers [20]. Given the progressive induction of the alternative pathway over time, in contrast to the classical pathway which is acutely and temporarily activated, the alternative pathway may represent an important source of PPO biomarkers given its progressive and persistent activation [21].

The purpose of this study was to determine whether serum and synovial fluid concentrations of alternative macrophage activation pathway mediators CHIT1 and CCL18 would differ in patients undergoing revision THR for PPO versus control patients without PPO being revised for another, noninfectious indication. Our hypothesis was that both CHIT1 and CCL18 would be significantly increased in the serum and synovial fluid of PPO versus control patients.

## Materials and Methods

This prospective case-control study was approved by the Hospital for Special Surgery Institutional Review Board (IRB). According to an a priori power analysis, 30 total patients were required to provide an α of 0.05 and β of 0.80. The primary outcome measure was differences in protein biomarker concentration, and the comparators were the osteolysis group and control group. The effect size change expected between groups was 5%. Patients undergoing revision metal-on-polyethylene THR at Hospital for Special Surgery were screened preoperatively from January 2013 to December 2014. Patients with active/prior infection (including positive intraoperative cultures), previous revision(s), metabolic/rheumatologic conditions, and/or medications affecting bone metabolism (such as steroids or bisphosphonates) were excluded. In addition, patients whose primary THR was performed for an indication other than osteoarthritis and/or within 6 months of revision THR were excluded. Study enrollment concluded when the predetermined study enrollment goal was met.

Sixty revision metal-on-polyethylene THR patients were screened preoperatively. Based on preliminary medical record and radiograph review, 26 patients were excluded prior to enrollment due to multiple revision surgeries (9), metabolic/rheumatologic condition (4), polyethylene acetabular component (3), primary hemiarthroplasty and multiple revision surgeries (3), metabolic/rheumatologic condition and multiple revision surgeries (3), polyethylene acetabular component and multiple revision surgeries (1), primary hemiarthroplasty (1), primary metal-on-metal THR (1), and revision THR performed 2 weeks after the primary THR (1). The remaining 34 patients were enrolled according to IRB protocol. Informed consent was obtained in the preoperative holding area. In total, 13 attending surgeons from the Adult Reconstruction and Joint Replacement Service contributed patients.

Preoperative serum was collected from the routinely inserted intravenous line by the anesthesiologist in the operating room prior to skin incision. The sample was collected in a tube with no additive and then placed on ice. Intraoperative synovial fluid was aspirated by the orthopedic surgeon immediately prior to arthrotomy (i.e., after skin incision) with a sterile syringe and 18-gauge needle. The synovial fluid sample was then placed on ice. In the laboratory, sample volumes were measured, aliquoted, and stored in a − 20°C freezer. Samples remained frozen until study enrollment was complete.

Following study enrollment, 4 additional patients were excluded due to intraoperative findings of loose implants (in patients with no osteolysis present on preoperative imaging and not suspected of having loose implants) (2), positive intraoperative cultures for infection (1), and zero synovial fluid volume (1). Thus, laboratory data was collected and analyzed from the samples from the remaining 30 patients: 20 in the “osteolysis” and 10 in the “control” group. Osteolysis patients underwent revision for PPO based on review of preoperative imaging and operative reports. Control patients had stable implants and were revised for recurrent dislocation (9) or mechanical symptoms (1).

The following data was obtained from medical record review for each patient: age, sex, date of primary THR, date of revision THR, laterality, past medical history, past surgical history (with particular attention to arthroplasty procedures for other joints), and medications. Operative notes were reviewed to determine the indications for revision THR, surgical procedure performed, and extent of PPO (e.g., whether implant was stable or loose). Final intraoperative cultures were followed and ensured to be negative. Preoperative imaging was reviewed: 14 patients had only radiographs, while the remaining 16 patients had advanced imaging as well (8 had only CT, 5 had only MRI, and 3 had both CT and MRI). All available imaging was reviewed to determine the presence of PPO, the THR implant type, and whether the femoral component was cemented or noncemented. No patients in this study had a cemented acetabular component. No patients in the control group had osteolysis on preoperative imaging. All patients in the osteolysis group had clear osteolysis in at least one zone in the femur and/or acetabulum, as defined by Gruen and Charnley, respectively [9, 11].

CHIT1 and CCL18 serum and synovial fluid concentrations were quantified via commercially available enzyme-linked immunosorbent assay (ELISA) kits (Circulex and R&D Systems, respectively) according to the manufacturer’s instructions. Each sample was performed in duplicate. The ELISA experiments were performed on 2 separate occasions by independent investigators. The results of these independent experiments yielded excellent agreement and identical conclusions; thus, the data presented represents average values from these 2 experiments. Concentrations are reported in nanomoles (nM).

Descriptive analyses of the study population consisted of reporting means (with standard deviations) for continuous variables and frequencies (with percentages) for categorical variables. Shapiro-Wilk tests were conducted and confirmed the non-normality of the data collected. As a result, nonparametric Mann-Whiney *U* tests were used to assess the differences in continuous variables between the osteolysis and control groups, while Fisher’s exact test was used to assess differences in categorical variables. Bivariate correlations between the serum versus synovial values were calculated using Pearson correlation coefficient. Statistical analysis was performed using SPSS 23 (IBM Corporation, Armonk, NY, USA).

## Results

Demographic data are shown in Table 1. There were no significant differences in age, sex distribution, or proportion of cemented femoral components. However, the osteolysis group had significantly older implants than the control group (*p* < 0.05).

**Table 1:** Demographic data for the control and osteolysis groups

	Control group	Osteolysis group
Number	10	20
Male/female	4:6	11:9
Mean age (years) ± standard deviation	63 ± 12	68 ± 11
Mean implant age (years) ± standard deviation	4.8 ± 6.0	14.0 ± 6.6
Cemented/noncemented femoral components	3:7	9:11

In the control group, revision surgery consisted of head/liner exchange (7) and acetabular component revision (3). In the osteolysis group, revision surgery consisted of component revision (6), acetabular component revision (6), head/liner exchange (5), and femoral component revision (3). Based on operative reports of patients in the control group, all implants were well fixed. Based on operative reports of patients in the osteolysis group, 10 had gross implant loosening (5 acetabulum, 5 femur), 6 had osteolysis around the femoral and acetabular components (but not grossly loose), 3 had osteolysis around the acetabular component (but not grossly loose), and 1 had osteolysis around the femoral component (but not grossly loose).

ELISA data are shown in Table 2. CHIT1 concentrations were significantly increased in the osteolysis versus control group’s synovial fluid and serum. CCL18 concentrations were significantly increased in the osteolysis versus control group’s synovial fluid but not serum.

**Table 2:** CHIT1 and CCL18 serum and synovial fluid concentrations in control and osteolysis groups based on ELISA data

	Control group	Osteolysis group	*P*
Serum CCL18	78 nM	66 nM	0.77
Serum CHIT1	39 nM	98 nM	< 0.01
Synovial fluid CCL18	180 nM	425 nM	< 0.01
Synovial fluid CHIT1	731 nM	3727 nM	< 0.01

Concentrations represent average values and are reported in nanomoles (nM)

There was a significant positive correlation between CHIT1 serum and synovial fluid values (Pearson correlation coefficient, 0.36; *p* < 0.05).

## Discussion

In this prospective case-control study, CHIT1 was validated as a potentially clinically useful synovial fluid and serum biomarker of PPO. While both CCL18 and CHIT1 concentrations were significantly increased in the osteolysis group’s synovial fluid, only CHIT1 was increased in the serum. Our synovial fluid findings are consistent with those of Koulouvaris et al., who demonstrated increased gene expression of CCL18 and CHIT1 in the periprosthetic tissues of patients undergoing revision THR for PPO versus controls (synovium excised during primary THR) [17]. These authors also demonstrated in vitro that CHIT1 expression was induced by both polymethyl methacrylate and metallic wear particles, as well as progressively induced during monocyte-to-macrophage differentiation [17]. Finally, in another recent study, CHIT1 synovial fluid concentrations were elevated to a comparable extent in patients with PPO as in our study [16]. Importantly, the current study utilized matched controls of patients undergoing revision for reasons other than PPO, helping consolidate the hypothesis that these potential biomarkers are specific for PPO. Taken together, these studies highlight the significantly increased local concentration of CHIT1 in response to particulate wear debris and sustained elevation in the setting of PPO.

This study has several important limitations. First, there is a potential impact of other joint replacements in the same patient. To minimize this effect, we excluded patients with known PPO or status postrevision arthroplasties for any other joints. Second, there were significant differences in implant age between the osteolysis and control groups. Several prior biomarker studies have selected matched outpatient controls, which is easier than matching operative cases. This approach has drawbacks, however, including that we would have been unable to correlate our serum and synovial fluid findings in the outpatient setting. Regardless, we are currently enrolling a matched outpatient control group for serum analysis that will serve as an additional control arm for our study. Finally, our osteolysis group had, by definition, severe PPO since undergoing revision surgery. This design is consistent with previous studies but raises the question of whether CHIT1 is similarly elevated earlier in the disease process that would ultimately determine its utility as a biomarker. To answer this question, a prospective study evaluating documented biomarkers in asymptomatic THR patients should be performed. In such a study, MRI screening of asymptomatic patients could be utilized to identify early cases of PPO not evident on radiographs.

Given the progressive and persistent induction of the alternative macrophage activation pathway over time, in contrast to the classical macrophage activation pathway’s acute and temporary activation in response to wear debris, we believe that the alternative pathway represents an important area for future PPO biomarker research. Interestingly, Chaganti et al. found no differences in serum levels of OPG or RANKL when comparing 15 patients scheduled for revision THR for osteolysis versus 15 matched outpatient controls [7]. These findings are also supported at the tissue level by Koulouvaris et al., who found no difference in RANKL expression in the periprosthetic tissues of osteolysis versus control patients (but did find significantly elevated levels of both CCL18 and CHIT1) [17]. Taken together, these findings highlight the critical importance of the alternative pathway in PPO, as well as in future PPO biomarker research.

It is interesting to note that the alternative macrophage activation pathway has been implicated in other diseases characterized by dysfunction of macrophage-mediated processes, such as Gaucher’s disease (a lysosomal storage disease) and sarcoidosis [4]. In these disease processes, alternative pathway activation occurs secondary to accumulation of poorly disposable material within macrophages. In Gaucher’s, a distinct alternative pathway gene expression profile has been described, including elevated CHIT1 and CCL18 [5, 6, 13]. Furthermore, in Gaucher’s, CHIT1 (and CCL18) serum levels are increased and have been clinically utilized as a biomarker to assess enzyme replacement therapy response [8, 15, 25]. Taken together, the clinical use of ELISA-determined serum CHIT1 (and CCL18) concentrations as a biomarker in Gaucher’s is an exciting potential lead for PPO biomarker research.

In conclusion, PPO remains the leading indication for revision THR. Delayed diagnosis is common and of clinical relevance to patients undergoing revision surgery. Our findings add to a growing body of evidence that the alternative macrophage activation pathway plays a central role in PPO pathogenesis. Furthermore, CHIT1 may represent a novel serum biomarker and potential therapeutic target for this disease.

## Electronic Supplementary Material

ESM 1 (XLSX 109 kb)

ESM 2 (PDF 1224 kb)

ESM 3 (PDF 1224 kb)

ESM 4 (PDF 1224 kb)

ESM 5 (PDF 1224 kb)

ESM 6 (PDF 1225 kb)

ESM 7 (PDF 1224 kb)

ESM 8 (PDF 1224 kb)
